# Authors’ Reply: Posttrial Withdrawal Ethics in the Healthy Ageing Ecosystem for People With Dementia (HAAL) Study

**DOI:** 10.2196/95516

**Published:** 2026-05-08

**Authors:** Giulio Amabili, Elvira Maranesi, Federico Barbarossa, Arianna Margaritini, Anna Rita Bonfigli, Fong-Chin Su, Chien-Ju Lin, Hsiao-Feng Chieh, Dianne Vasseur, Henk Herman Nap, Yeh-Liang Hsu, Dorothy Bai, Roberta Bevilacqua

**Affiliations:** 1 Scientific Direction IRCCS INRCA Ancona Italy; 2 Medical Device Innovation Center National Cheng Kung University Tainan City Taiwan; 3 Vilans | The Dutch Centre of Expertise for Long Term Care Utrecht, Utrecht The Netherlands; 4 School of Gerontology and Long-Term Care College of Nursing Taipei Medical University Taipei Taiwan

**Keywords:** older people, dementia, dashboard, technology for older adults, digital health, eHealth, innovation in health care

## Letter to the Editor

We are grateful for the thoughtful and constructive reflections [1] on posttrial responsibilities toward people living with dementia and their caregivers in response to our study [2]. Below, we explain how the issues raised were addressed in the original protocol and within the study.

First, we fully acknowledge the growing ethical relevance of posttrial access and managing potential dependency effects when time-limited technologies are withdrawn, especially for vulnerable people.

It is important to clarify that the Healthy Ageing Ecosystem for People with Dementia (HAAL) technologies are not assistive devices in the conventional sense, which are usually defined and regulated in close connection with disability conditions, aiming to substitute or compensate for impaired functions [3]. HAAL belongs instead to the domain of ambient assisted living (AAL) technologies: interconnected digital ecosystems designed to support self-management and care coordination without replacing human care activities [4]. In this AAL context, the most strongly felt ethical issue lies in preventing subtle forms of technological dependency where overreliance on digital monitoring and alerts might exacerbate isolation and perceived loneliness rather than enhance safety and social connection. In fact, one key driver to their acceptance is precisely the recognition that they cannot replace a human presence and may even increase solitude if positioned as such. For this reason, HAAL placed fundamental emphasis on training in eHealth and technological literacy as a preventive measure before system use, implemented from the outset. This was designed not only to enable participants to use the technology correctly, but to foster realistic representations and expectations of what AAL ecosystems can and cannot achieve, preventing overreliance and so dependency [4]. Through this approach, people with dementia and their caregivers were empowered to integrate HAAL into broader care strategies rather than viewing it as a standalone or substitutive solution.

As regards the study conduct, participants were informed from the outset that the HAAL ecosystem was a temporary research intervention with system removal at the end of the 3-month period, as clearly outlined in consent processes and scheduled follow-up visits. The fixed duration was strategically chosen to evaluate safety, usability, and acceptability during early feasibility testing while minimizing risks of long-term dependency formation. Moreover, research equipment could not be left to participants due to strict funding rules and public procurement procedures, which prohibit the indefinite allocation of project-funded hardware and software licenses once the grant period concludes. We also provided extended counselling sessions, practical information about community and health care alternatives, and caregiver guidance to help sustain positive strategies independently of device availability. Finally, once enrolled through a study center, participants remained integrated within the broader care and research network, maintaining clear channels for communicating evolving needs and for potential involvement in future innovation projects.

We concur on the need to thoughtfully adapt clinical ethics concepts like continued access and posttrial responsibilities to the specific realities of research, where interventions are often provided through temporary prototypes rather than permanent tools. In upcoming projects, and considering time and budget constraints, we plan to include explicit withdrawal-impact assessments among the outcome measures, and to reinforce our exit strategies.

## Abbreviations

AAL: ambient assisted living
HAAL: Healthy Ageing Ecosystem for People with Dementia
